# Single-cell RNA-Seq analysis reveals dynamic trajectories during mouse liver development

**DOI:** 10.1186/s12864-017-4342-x

**Published:** 2017-12-04

**Authors:** Xianbin Su, Yi Shi, Xin Zou, Zhao-Ning Lu, Gangcai Xie, Jean Y. H. Yang, Chong-Chao Wu, Xiao-Fang Cui, Kun-Yan He, Qing Luo, Yu-Lan Qu, Na Wang, Lan Wang, Ze-Guang Han

**Affiliations:** 1 Key Laboratory of Systems Biomedicine (Ministry of Education) and Collaborative Innovation Center of Systems Biomedicine, Shanghai Center for Systems Biomedicine, Shanghai Jiao Tong University, 800 Dongchuan Road, Shanghai, 200240 China; 2 Key Laboratory of Computational Biology, CAS-MPG Partner Institute for Computational Biology, 320 Yueyang Road, Shanghai, China; 3 School of Mathematics and Statistics, The University of Sydney, Sydney, Australia; 4 Shanghai-MOST Key Laboratory for Disease and Health Genomics, Chinese National Human Genome Center at Shanghai, Shanghai, China

**Keywords:** Liver stem/progenitor cells, Single-cell RNA-Seq, Developmental trajectory, Cholangiocyte, Fate decision

## Abstract

**Background:**

The differentiation and maturation trajectories of fetal liver stem/progenitor cells (LSPCs) are not fully understood at single-cell resolution, and a priori knowledge of limited biomarkers could restrict trajectory tracking.

**Results:**

We employed marker-free single-cell RNA-Seq to characterize comprehensive transcriptional profiles of 507 cells randomly selected from seven stages between embryonic day 11.5 and postnatal day 2.5 during mouse liver development, and also 52 Epcam-positive cholangiocytes from postnatal day 3.25 mouse livers. LSPCs in developing mouse livers were identified via marker-free transcriptomic profiling. Single-cell resolution dynamic developmental trajectories of LSPCs exhibited contiguous but discrete genetic control through transcription factors and signaling pathways. The gene expression profiles of cholangiocytes were more close to that of embryonic day 11.5 rather than other later staged LSPCs, cuing the fate decision stage of LSPCs. Our marker-free approach also allows systematic assessment and prediction of isolation biomarkers for LSPCs.

**Conclusions:**

Our data provide not only a valuable resource but also novel insights into the fate decision and transcriptional control of self-renewal, differentiation and maturation of LSPCs.

**Electronic supplementary material:**

The online version of this article (10.1186/s12864-017-4342-x) contains supplementary material, which is available to authorized users.

## Background

The two major epithelial cell types of liver, hepatocytes and cholangiocytes, are differentiated from hepatoblasts during embryonic liver development [1–3]. Liver stem/progenitor cells (LSPCs) have been suggested to exist in fetal and adult liver and are generally defined as cells with the potential to differentiate into both hepatocytes and cholangiocytes [4]. Hepatoblasts are considered a type of LSPC during liver development; however, liver development involves many different cell types derived from endoderm and mesoderm and their reciprocal interactions. Thus, it is possible that fetal LSPCs could exhibit more complex genetic programs and developmental controls [5].

Previous studies involving the cell fate determination of LSPCs relied on isolation with surface membrane proteins such as EpCAM [6, 7], DLK1 [8], E-cadherin [9], CD13 [10, 11], and CD133 [11]. However, the cell fate determination of LSPCs isolated from embryonic livers, by employing different surface markers is controversial, reflecting the complexity of liver organogenesis and the heterogeneity of LSPCs [4]. Liver development is a dynamic process with the possibility of changing cell markers within LSPC populations. Thus, isolation of LSPCs based on the a priori knowledge about the limited surface markers may restrict the recognition of LSPCs and their functional features to some extent. The best way to perform the clonogenicity and repopulation assays is to assess the differentiation potential of single cells from embryonic livers; however, the practical difficulty of in vivo or ex vivo experiments hinders observation of the trajectory of cell lineage differentiation and maturation of these naïve LSPCs at single-cell resolution.

Recently, the development of single-cell sequencing-based technology has provided a unique chance to address many longstanding questions, such as cell lineage relationships and heterogeneity in a given cell population [12–14]. Single-cell transcriptomic analysis, such as RNA-Seq, would supplant the coarse notions of the marker-based cell types and uncover new cell types by the unbiased sampling of single cells [15]. For liver research, the gene expression profiles of zonation and spatial division of hepatic lobule in adult mouse liver were revealed at single-cell resolution [16], and multi-lineage communication is also shown to be important for human liver bud development [17]. Currently, the definition and molecular state of LSPCs during liver development are still obscure, and the developmental trajectories of LSPCs, including self-renewal, differentiation and maturation, are not fully understood at single-cell resolution.

To address the above questions, in this study, we applied single-cell RNA-Seq and quantitative RT-PCR (qPCR) to analyze ~ 800 single cells from eight different stages during mouse liver development. The transcriptomic analysis of LSPCs reconstructed their stepwise differentiation and maturation process at single-cell resolution. In addition to cell surface molecules, we also uncovered the contiguous but discrete genetic control by transcription factors and signaling pathways. To further understand the fate decision and differentiation process of LSPCs, we also analyzed the single-cell transcriptomic profiles of Epcam-positive cholangiocytes. Our single-cell transcriptomic analysis of LSPCs during mouse liver development provides insights into the transcriptional control of their self-renewal, differentiation and maturation and is a useful resource for future research, including research on isolation methods designed for LSPCs.

## Results

### Overview of single-cell qPCR and RNA-Seq of developing mouse livers

**Fig. 1:**
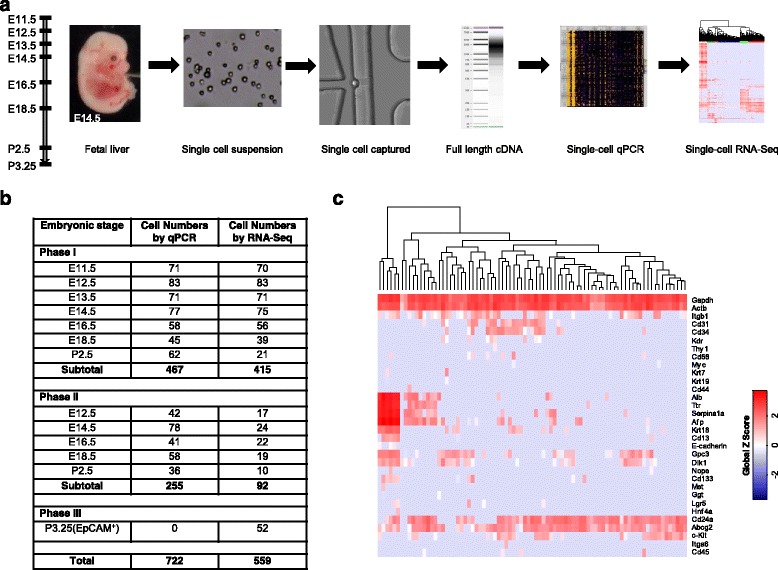
Overview of single-cell analysis of developing mouse fetal livers. **a** Experimental workflow. **b** Statistics of the single cells analyzed in this study. **c** Single-cell qPCR analysis of mouse fetal liver cells, with E12.5 as an example

To comprehensively understand the transcriptional program during liver development, we carried out single-cell transcriptomic analysis, including qPCR, on 722 cells and RNA-Seq on 559 cells derived from mouse fetal livers at eight developmental stages, including embryonic day (E) 11.5, 12.5, 13.5, 14.5, 16.5, 18.5 and postnatal day (P) 2.5 and P3.25 (Fig. 1a-b). We first randomly selected 467 single cells and then assessed them via single-cell qPCR with genes related to cell types and liver development (Fig. 1c and Additional file 1: Figure S1). We observed that hepatic marker genes such as *Afp*, *Alb*, *Ttr* and *Serpina1a* were highly expressed in some cells from E11.5 to E16.5 livers, which were later identified as hepatoblasts. However, a similar gene expression pattern was rarely observed in single cells from E18.5 and P2.5 livers (Additional file 1: Figure S1). After removing low quality libraries, we performed RNA-Seq on 415 single cells using the same cDNA libraries as qPCR. We proposed the molecular patterns for putative LSPCs after analysis of these cells and then collected 255 single cells from another batch of fetal livers as biological replicates, and 92 single cells were chosen for RNA-Seq (Fig. 1b). We also used flow cytometry to isolate Epcam^+^ cells from P3.25 livers, which were likely to be cholangiocytes [7, 18], and then sequenced 52 these Epcam^+^ single cells (Fig. 1b).

In this study, the median mapping rates of sequencing reads within each developmental stage ranged from 57% to 78%. The median numbers of unique mapped reads ranged from 1.1 to 3.8 million per cell. The median numbers of genes detected with confidence of fragments per kilobase of exon model per million (FPKM) > 1 ranged from approximately 3000 to 6000 for all stages except Epcam^+^ cells from P3.25 livers, which only showed a median number of around 2000 genes despite similar sequencing depth and mapping rate (Additional file 1: Figure S2a and Additional file 2: Table S1). The decreased number of genes expressed in Epcam^+^ cells from P3.25 livers could be due to their more differentiated status. We introduced ERCC RNA Spike-ins as technical controls, and high correlation coefficients among single cells at each stage based on the 92 Spike-ins were observed (Additional file 1: Figure S2b), indicating low technical noise in our data. We further quantitatively evaluated the correlation between RNA-Seq and qPCR data from the same single cells, and they were positively correlated with each other (Additional file 1: Figure S2c). Here, the median correlation coefficients between single-cell RNA-Seq and qPCR were approximately 0.9 for all stages (Additional file 1: Figure S2c).

### Identification of LSPCs in developing mouse livers via marker-free transcriptomic profiling

**Fig. 2:**
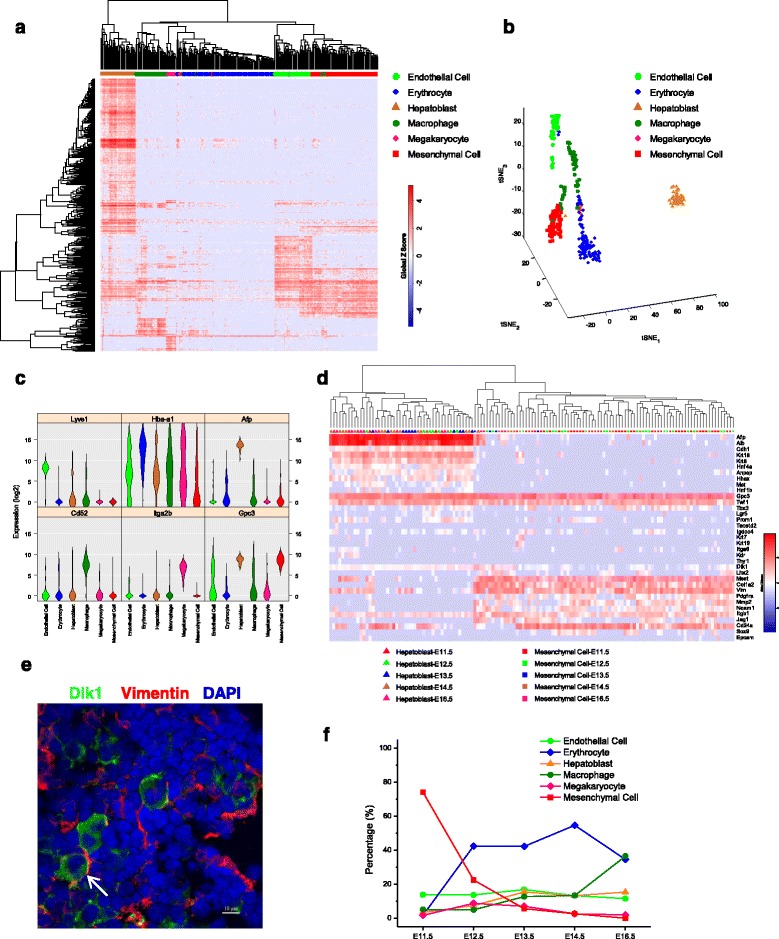
Decomposition of the constituent cell types in mouse fetal livers. **a** Hierarchical clustering showing cell types identified. **b** Visualization of cell types using t-SNE. **c** Violin plot of six marker genes. **d** Comparison of the gene expression profiles between hepatoblasts and mesenchymal cells with selected marker genes. **e** Expression of Dlk1 and vimentin in E11.5 mouse liver shown by immunofluorescence assay. A cell co-expressing Dlk1 and vimentin was indicated by white arrow. Scale bar, 10 μm. More detailed figures are shown in Additional file 1: Figure S4a-b. **f** The temporal changes in the proportions of the six cell types

Limited markers may lead to the incorrect identification of cell populations, and single-cell transcriptomic profiling facilitates ab initio cell-type characterization. Because a very large portion of E18.5 and P2.5 cells were mature hepatocytes (Additional file 1: Figs. S1, S5), we focused on single cells from E11.5 to E16.5 for cell type identification. To reduce the disturbance in the gene expression-based cell clustering analysis, the transcripts severely corrupted by technical noise were filtered via a statistical model (Additional file 1: Figure S2d). Subsequently, hierarchical clustering (HC) enabled decomposition of these cells into six groups which were later confirmed as endothelial cells, erythrocyte, hepatoblast, macrophage, megakaryocyte and mesenchymal cells (Fig. 2a and Additional file 1: Figure S3a-b), where the classification also could be clearly visualized with t-distributed stochastic neighbor embedding (t-SNE) plot (Fig. 2b).

Among the six groups, one group was classified as the endothelial lineage, while three were assigned to myeloid lineages of the hematopoietic system, including erythrocytic, megakaryocytic, monocytic or macrophagic (likely Kupffer cells) lineages based on specific markers (Fig. 2c and Additional file 1: Figure S3b) and the Gene Ontology (GO) enrichment analysis (Additional file 1: Figure S3c), consistent with the fact that fetal liver is the main hematopoietic organ during this developmental period. These single cells expressed known lineage-specific marker genes; for example, endothelial cells express *Lyve1* and *Kdr*; erythrocytes highly express *Hba-a1* and *Hbb-bt*; macrophages express *Ptprc* (*Cd45*), *Cd68* and *Cd52*; and megakaryocytes express *Itga2b* and *Itgb3*.

One of the remaining two groups belonged to hepatoblasts that express hepatic markers such as *Afp* and *Alb*, stem/progenitor-related genes such as *Gpc3* and *Dlk1* (Fig. 2c and Additional file 1: Figure S3a), and genes related to liver functions such as lipid metabolism and blood coagulation (Additional file 1: Figure S3c). Interestingly, the other group expressed some stem/progenitor-related genes, such as *Gpc3*, *Dlk1*, *Sox9* and *Sox11*, but had no or low expression of genes related to hepatic lineages (Additional file 1: Figure S3b).

As known, the liver bud is expanding in septum transversum mesenchyme at E11.5 stage, so we speculated that this un-identified group may be related to the mesenchymal phenotype. By checking the expression profiles of certain marker genes closely related to LSPCs and mesenchymal cells (Fig. 2d), we figured out the distinct signatures that can distinguish hepatoblasts from mesenchymal cells. Significantly, hepatoblasts highly expressed many hepatic lineage-specific markers, such as *Afp*, *Alb*, *Hnf4a*, *Krt18*, *Krt8*, *Hnf1b*, *Hhex* and *Met* (*c-Met*), as well as *Anpep* (*Cd13*) and *Cdh1* (E-cadherin), while the un-identified group expressed significantly higher levels of mesenchymal-related marker genes such as *Vim*, *Col1a2*, *Mest*, *Mmp2*, *Pdgfra*, *Ncam1* and *Lhx2*. We thereby named the group as mesenchymal cells. The interesting thing is that *Dlk1*, a marker of hepatic stem cells, was expressed in the group classified as mesenchymal cells. Here we thus employed immunofluorescence assay and confirmed the existence of such cells co-expressing both Dlk1 and vimentin, a mesenchymal marker (Fig. 2e and Additional file 1: Figure S4a-b).

Epcam is a marker closely related to the differentiation of LSPC into cholangiocytes [4, 18]. Our single-cell RNA-Seq data showed that *Epcam* expression was detected in 1 of 2 cells of E11.5 hepatoblasts and 5 of 43 cells of E11.5 mesenchymal cells, not detected in E12.5 ~ E14.5 cells, but was detected again in 2 of 12 cells of E16.5 hepatoblasts (Fig. 2d). This temporal change of *Epcam* expression indicated our single-cell RNA-seq data was consistent with the previous observation via immunofluorescence assay or flow cytometric analysis [7]. As single-cell RNA-Seq may suffer drop-out issue, here we selected the 8 cells with *Epcam* transcript and 8 cells without *Epcam* transcript form E11.5 and E16.5 for further qPCR validation. For cDNA library before Nextera amplification, *Epcam* expression was only detected in 1 and 2 cells by microfluidic-based and tube-based qPCR, respectively; while for cDNA library after Nextera amplification, *Epcam* expression was detected in 4 cells by tube-based qPCR (Additional file 1: Figure S4c). The comparison of the two approaches indicated that our single-cell RNA-Seq data at the current sequencing depth was more sensitive than qPCR in detecting low expressed genes, which also showed consistence with the results in Additional file 1: Figure S2c. The reason that we didn’t detect *Epcam* expression in many cells in these developing livers is probably because *Epcam* is expressed in a small fraction of LSPCs, where the marker-free approach was difficult to detect them.

We then characterized the distribution of the six groups at each stage. Interestingly, the fetal livers from E11.5 to E16.5 all had six cell types despite different proportions (Fig. 2f). The proportion of erythrocytes increased from E11.5 to E14.5 and then decreased at E16.5, and a stepwise decrease in mesenchymal cells and a slight increase in hepatoblasts from E11.5 to E16.5 were observed.

### Dynamic developmental process of LSPCs at single-cell resolution

To decipher the dynamic developmental process of LSPCs during liver development, we performed gene set enrichment analysis (GSEA) of hepatoblasts along the developmental stages. The gene expression pattern of hepatoblasts from E12.5 to E14.5 had no statistically meaningful differences, but the comparison of E14.5 and E16.5 hepatoblasts revealed that the cell cycle and mitosis-related genes were significantly enriched in the E14.5 stage (Additional file 1: Figure S5a). This finding suggested that the transition from E14.5 to E16.5 may be the critical differentiation switch for hepatoblasts via cell division.

**Fig. 3:**
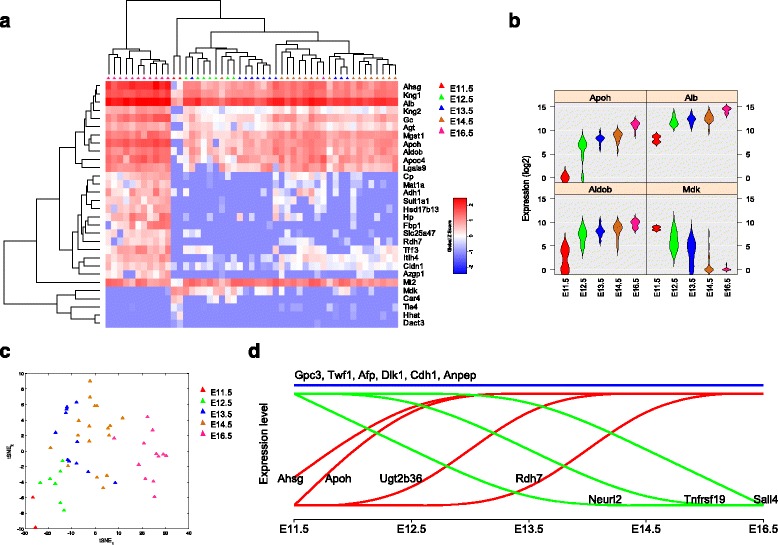
Dynamic developmental process of mouse LSPCs at single-cell resolution. **a** HC analysis using genes that were differentially expressed among the five developmental stages. **b** Violin plot of selected genes related to hepatoblasts development. **c** Developmental track of hepatoblasts was shown by t-SNE plot. **d** Dynamic developmental process of hepatoblasts with representative gene expression patterns shown

We then analyzed all hepatoblasts from E11.5 to E16.5 livers to construct the landscape of the dynamic developmental processes. Analysis of variance (ANOVA) was first applied to rank the genes that were differentially expressed across the five developmental stages, and the top 30 genes (Additional file 3: Table S2) were used for the developmental track construction. The gradually up-regulated genes included *Apoh*, *Ahsg*, *Alb*, *Kng2*, *Adh1* and *Aldob*, which are specific for hepatocyte function; whereas the genes that were decreased stepwise included *Mdk* that is highly expressed in mid-gestation [19], and *Hhat* (hedgehog acyltransferase) that is required for SHH signaling which is closely related to embryonic development, liver regeneration and liver cancer stem cells [20–23] (Fig. 3a-b and Additional file 1: Figure S5b). In the developmental track, it is clear that E12.5 ~ E14.5 hepatoblasts were closely related in a continuous manner (Fig. 3c). The stepwise gene expression profile changes also provided insights into the transcriptional programs of hepatoblast differentiation and maturation (Fig. 3d).

Theoretically, hepatoblasts will differentiate into hepatocytes and cholangiocytes, but the hepatoblasts from E11.5 to E16.5 only exhibited stepwise increased expression of hepatic-related proteins without expressing biliary markers. Are these cells indeed hepatoblasts or differentiated immature hepatocytes? To figure this out, we compared these embryonic hepatoblasts with P2.5 hepatic cells. Only one hepatic cell from the P2.5 stage was closely related to E16.5 hepatoblasts, and all others were distinct from hepatoblasts, where they had low or no expression of stem/progenitor-related markers, such as *Gpc3*, *Dlk1* and *Afp*, but higher expression of hepatocyte-related genes, such as *Fbp1*, *Mat1a* and *Sult1a1* (Additional file 1: Figure S5c-d). The data indicated that the majority of hepatic cells from P2.5 livers were hepatocytes, although there were a few hepatoblasts, possibly representing precursors for oval cells within adult livers. Here, our data indicated that hepatoblasts in E11.5 ~ E16.5 are authentic hepatoblasts.

### LSPC differentiation into cholangiocytes

As hepatoblasts are the bi-potential progenitors for both hepatocytes and cholangiocytes, it is important to reveal the cell fate decision of LSPC differentiation into cholangiocytes. LSPCs are generally believed to co-express hepatocyte and cholangiocyte markers, and we checked such a possibility in these single liver cells from E11.5 to E16.5. Only 4 cells from E11.5, E12.5 and E14.5 livers co-expressed hepatocyte-specific markers, such as *Krt8* and *Krt18*, and cholangiocyte-specific markers, such as *Krt7* and *Krt19* (Fig. 2d). The results indicated that only a minority of hepatoblasts from mouse fetal livers co-express hepatocyte and cholangiocyte markers at this developmental period. Our randomly selected single cells contain few cells showing markers of cholangiocytes, probably due to the asymmetric lineage fate of hepatoblast differentiation into hepatocyte and cholangiocyte. We thus employed flow cytometry to enrich the relative rare mouse cholangiocytes using the well-known marker Epcam [6, 7, 18], which will enable single-cell transcriptomic comparison between cholangiocytes and hepatoblasts and facilitate understanding the fate-decision stage for differentiation into cholangiocytes. We obtained 52 Epcam^+^ single cells from P3.25 fetal livers and sequenced them.

**Fig. 4:**
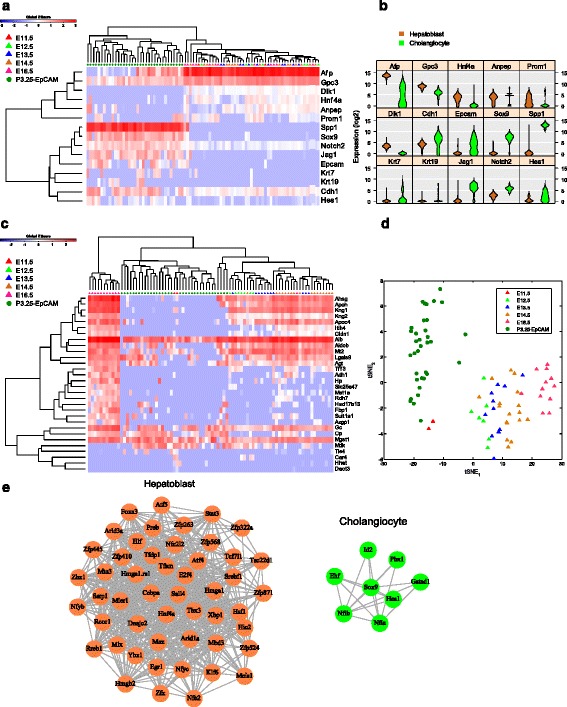
Distinct transcriptomic features between hepatoblasts and cholangiocytes. **a** HC showing the heterogeneity of gene expression of some selected marker genes in hepatoblasts and cholangiocytes. **b** Violin plot of selected marker genes in hepatoblasts and cholangiocytes. Comparison of the gene expression profiles of P3.25 cholangiocytes and hepatoblasts from different stages by HC (**c**) and t-SNE plot (**d**) are shown. **e** Transcription factors covariance networks of hepatoblasts and cholangiocytes. Each node represents a TF, and each edge represents correlation coefficient higher than 0.35. The two networks are colored to discriminate TFs specifically related to hepatoblasts and cholangiocytes

We compared the expression of LSPC-related markers between hepatoblasts and the P3.25 Epcam^+^ cells (Fig. 4a-b). Some genes such as *Hnf4a*, *Dlk1*, *Anpep* and *Prom1* (*Cd133*) were highly expressed in hepatoblasts, but not in the Epcam^+^ cells. Significantly, *Gpc3* expression was maintained in all Epcam^+^ cells, whilst *Afp* was only expressed in about half of the Epcam^+^ cells. Another interesting phenomenon is that the expression level of *Cdh1* was increased in the Epcam^+^ cells, suggesting high E-cadherin level could be a putative marker for cholangiocyte isolation. The genes highly and specifically expressed in the Epcam^+^ cells included *Sox9* and *Spp1*, which are well-documented markers for cholangiocytes. The expression of *Krt7* or *Krt19* emerged in some of the Epcam^+^ cells, but not in E11.5 ~ E16.5 hepatoblasts. We also checked the expression pattern of marker genes from Fig. 4a in P3.25 Epcam^+^ cells and hepatoblasts to hESC-derived cholangiocytes and hepatoblasts (hESC-Chol and hESC-HB) [24]. Seven genes from Fig. 4a were found to be differently expressed between hESC-Chol and hESC-HB, and similar changing expression patterns were also observed between mouse cholangiocytes and hepatoblasts (Additional file 1: Figure S6a). The expression of *DLK1* was decreased in hESC-Chol in comparison with hESC-HB, also consistent with disappearing expression of *Dlk1* in single cells of mouse cholangiocytes (Additional file 1: Figure S6b). There was one exception that *AFP* expression was increased in hESC-Chol, while single cells of mouse cholangiocytes showed heterogeneous *Afp* expression pattern (Additional file 1: Figure S6b). This is probably because the hESC derived cholangiocytes are not exactly the same as their in vivo counterparts. The collective data indicated that these Epcam^+^ cells are cholangiocytes.

Currently it is still not clear when LSPCs are fated to hepatocytes or cholangiocytes. To decipher the issue, we compared transcriptomic feature of these P3.25 Epcam^+^ cholangiocytes with hepatoblasts from E11.5 ~ E16.5 using the differentially expressed transcripts among these stages (see Fig. 3). Interestingly, the gene expression profiles of P3.25 cholangiocytes were more closely related to that of E11.5 hepatoblasts rather than other hepatoblasts from later developmental stages (Fig. 4c-d). The resembled gene expression profiles between cholangiocytes and early hepatoblasts hint that hepatoblasts may be fated to cholangiocytes at an early stage. However, this single-cell genomics-derived model still needs further validation, especially solid evidence from well-designed lineage tracing experiments employing reliable markers.

We further focused on the signaling pathways related to liver development, including the Wnt/β-catenin, Notch, TGF-β and Hedgehog pathways. The results showed that *Jag1*, *Notch2* and *Hes1* were significantly (*p* < 0.01) higher expressed in cholangiocytes, suggesting that Notch activation was associated with cholangiocytes differentiation (Fig. 4a-b), consistent with previous results [18].

As known, Transcription factors (TFs) play important roles in liver development. Therefore, we screened TFs that were differentially expressed between hepatoblasts and cholangiocytes and found more TFs were specifically or highly expressed in hepatoblasts (Additional file 4: Table S3), suggesting stem/progenitor cells with higher plasticity could maintain a high self-renewal and differentiation capability under complex regulatory conditions. We then performed expression covariance network analysis and constructed two TF covariance networks specific to hepatoblasts and cholangiocytes (Fig. 4e). Within the hepatoblast-specific network, the TFs that had significant correlations with more TFs included Atf4, Tfam, Hmga1, Maz, Sall4, Mbd3, Tfdp1, Hnf4a, Ssrp1 and E2f4, some of which are required for liver functions or regulation of cell self-renewal and differentiation. For examples, zinc finger transcription factor Sall4 controls the cell fate decision of hepatoblasts and serves as a marker for progenitor subclass of hepatocellular carcinoma [25, 26]. For the cholangiocyte-specific network, such TFs included Sox9, Nfib, Nfia, Ehf, Id2, Gatad1, Pbx1 and Hes1, some of which are known to be involved in various stem cell-related signaling pathways. For example, Hes1 is closely related to Notch pathway, while the HMG-box transcription factor Sox9 has been shown to be related to progenitor status and differentiation of cholangiocytes [27, 28]. The TF covariance analysis not only supports that some well-studied TFs may play critical roles in LSPC differentiation but also provides candidate TFs for future investigation.

### Assessment and prediction of LSPC biomarkers

**Fig. 5:**
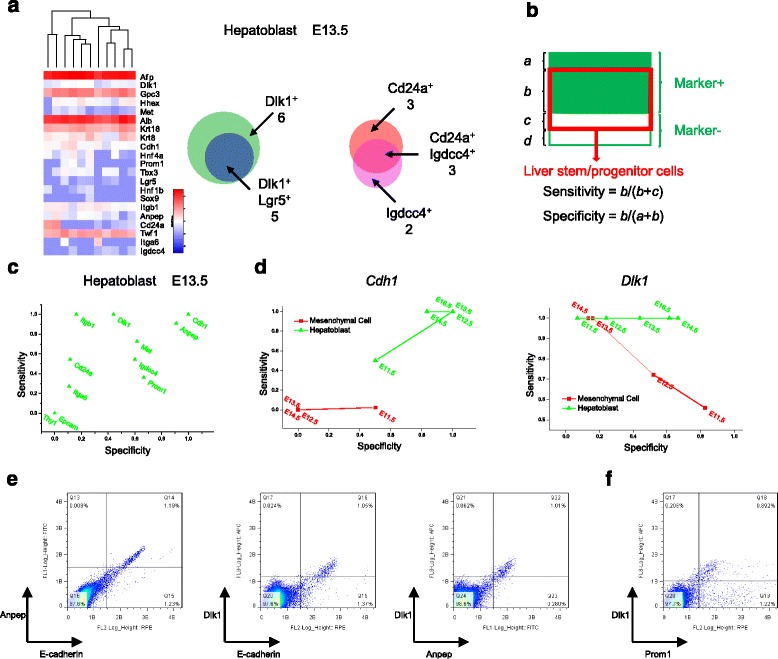
Assessment of LSPC biomarkers. **a** Heterogeneity of gene expression in LSPCs. Co-expression analysis of representative gene pairs in E13.5 hepatoblasts are shown. **b** Definition of isolation sensitivity and specificity based on randomly selected single cells with cell type information inferred from global transcriptional profiles. **c** Sensitivity vs. specificity plot of 11 selected markers for E13.5 hepatoblasts. **d** Sensitivity vs. specificity plot of LSPC isolation using *Cdh1* and *Dlk1*. **e-f** Co-expression analysis of E-cadherin, Anpep and Dlk1, and Dlk1 and Prom1 in E14.5 fetal livers via flow cytometry. Representative images from two replicative reactions for each condition are shown

The isolation of LSPCs in previous studies generally relied on limited surface markers. The gene expression patterns of commonly used markers confirmed the heterogeneity within LSPCs (Fig. 5a and Additional file 1: Figure S7a). We checked the combined expression profiles of some representative genes. As an example, the marker-positive cells with the gene pairs such as *Dlk1* vs. *Lgr5* and *Cd24a* vs. *Igdcc4* (*Nope*) were not identical in hepatoblasts at E13.5 although overlapped (Fig. 5a). The results indicated that LSPCs isolated with different markers may represent overlapping but not identical LSPC pool. As our single cells were randomly selected from fetal livers, systematic assessment of the sensitivity and specificity of isolation markers for LSPCs could be performed according to the proposed approach (Fig. 5b).

As the phenotypes of LSPCs are dynamically changing during the developmental process, we separately analyzed 11 marker genes commonly used for cell isolation in hepatoblasts at each stage (Fig. 5c and Additional file 1: Figure S7b). *Cdh1* exhibited the best sensitivity and specificity for hepatoblast isolation from E12.5 to E16.5 livers (Fig. 5d), followed by *Anpep* and *Prom1* (Additional file 1: Figure S7c). *Dlk1* was expressed in both hepatoblasts and mesenchymal cells at early stage, where its specificity gradually increased from E11.5 to E16.5 for hepatoblasts and decreased from E11.5 to E14.5 for mesenchymal cells (Fig. 5d). The specificity of *Igdcc4* for hepatoblasts gradually increased from E11.5 to E16.5, despite its decreased sensitivity (Additional file 1: Figure S7c). We also checked *Gpc3*, and the specificity decreased in mesenchymal cells but increased in hepatoblasts over time (Additional file 1: Figure S7c). Our data provide a systematic evaluation of the isolation markers for LSPCs, which is helpful for evaluating the reliability of previously isolated LSPCs and for predicting isolation markers for further validation. For example, *Gcgr* and *Cdhr2* are predicted to be isolation markers for hepatoblasts (Additional file 1: Figure S7c).

To validate the results, we performed flow cytometric analysis of mouse fetal liver cells from different stages with antibodies against E-cadherin, Anpep, Dlk1 and Prom1. The co-expression of E-cadherin, Anpep and Dlk1 was observed in E12.5 ~ E16.5 fetal liver cells (Fig. 5e and Additional file 1: Figure S8a-b), consistent with our single-cell RNA-Seq data. However, there was inconsistency in regard to Dlk1 expression in E12.5 fetal livers. Flow cytometry indicated that Dlk1^+^ cells almost perfectly overlapped with E-cadherin^+^ cells (Additional file 1: Figure S8a), whereas RNA-Seq data showed that *Dlk1* expression could not distinguish between hepatoblasts and mesenchymal cells at E12.5 stage and that *Cdh1* is a specific marker for hepatoblasts (Fig. 5d). This finding suggested that protein levels are not always consistent with transcript levels. Flow cytometric analysis also revealed that only a portion of the Prom1^+^ cells from E14.5 and E16.5 fetal livers were Dlk1-positive (Fig. 5f and Additional file 1: Figure S8c). In general, both single-cell RNA-Seq and flow cytometry supported E-cadherin, Anpep and Dlk1 as appropriate biomarkers for isolation of LSPCs, while Prom1 may slightly differ from the above three markers (Figs. 2d, 5e-f and Additional file 1: Figs. S7c, S8).

## Discussion

There have been DNA microarrays or RNA-Seq analyses of fetal livers from different stages [29, 30], but analysis based on average gene expression signals from different constituent cell types makes it difficult to ascribe the gene expression changes to a specific cell type. Single-cell analysis thus facilitates a more accurate identification of gene expression changes related to LSPCs. In this study, we employed single-cell qPCR and RNA-Seq to systematically re-visit the developmental process of mouse fetal livers, and the marker-free approach based on global transcriptional profiles enabled more reliable identification of the constituent cell types in fetal livers. Our data support the systematic assessment and prediction of markers for LSPC isolation, and reconstructed the developmental track of hepatoblasts at single-cell resolution.

**Fig. 6:**
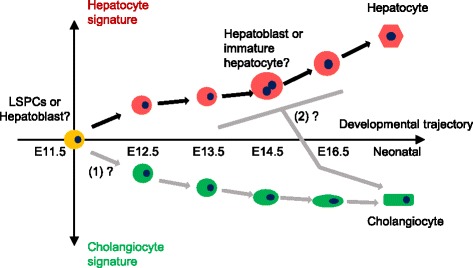
The proposed schematic diagram of the fate decision and differentiation of LSPCs. From E11.5 to neonatal, the increased expression of hepatic- or biliary-related genes indicates an elevated hepatocyte or cholangiocyte signature in LSPCs. Black arrows indicate the observed developmental processes in our data, while grey arrows indicate the putative developmental steps. “(1)” and “(2)” denote two possible stages where the fate decisions of LSPCs occur

Hepatoblasts will theoretically differentiate into both hepatocytes and cholangiocytes, and when they are fated to or differentiate into the two lineages is an important question still unanswered. Currently, it is found that, at around E13.5 ~ E16.5, hepatoblasts adjacent to portal veins are induced by Notch, TGF-β or other signals from nearby mesenchymal or endothelial cells to form the ductal plate and later differentiate into intra-hepatic ductal cells [18, 27]. This implies that hepatoblasts are not pre-fated to hepatic or biliary lineage but that their locations will decide their fates. Our single-cell data showed that the changes in hepatoblasts during this period are mainly related to stepwise increased hepatic functional gene expression (Figs. 3, 6). The cell cycle- and mitosis-related genes were significantly enriched in E14.5 hepatoblasts (Additional file 1: Figure S5a), suggesting that these cells may be induced by an inherited program or exterior environment to initiate differentiation via cell division (Fig. 6), consistent with the previous model.

It was found that the gene expression profiles of single Epcam^+^ cholangiocytes from P3.25 livers were more closely related to E11.5 hepatoblasts rather than other later hepatoblasts, implying that LSPCs may have their fate decision for cholangiocytes differentiation at an early stage (Fig. 4). The differentiation of hepatoblasts into hepatocytes and cholangiocytes is asymmetrical, and the proportion of hepatic-fated hepatoblasts appeared to be much higher than ductal-fated cells, which could explain the reason why these hepatoblasts derived from our random approach only showed differentiation directions towards hepatocytes. Our single-cell transcriptomic analysis thus provides insights about the fate decision stage of hepatoblasts.

Our data showed the existence of some cells co-expressing both hepatic and mesenchymal markers during mouse liver development. In this study, the immunofluorescence assay confirmed there were cells co- expressing both Dlk1 and vimentin in E12.5 fetal livers. This finding is consistent with previous observations of liver cells that co-express epithelial and mesenchymal markers [31, 32]. The results suggested the possible involvement of epithelial-mesenchymal transition (EMT) or mesenchymal-epithelial transition (MET), consistent with recent observation of EMT-MET during the differentiation of hESC into hepatocytes [33], but this needs further evidences such as lineage tracing experiments.

## Conclusions

In summary, our data provides a useful resource describing LSPCs at single-cell resolution during mouse liver development. Our marker-free approach provides insights into the reliable isolation of LSPCs and their developmental track, and analysis of single cholangiocytes further facilitates understanding of cell fate decision, differentiation and regulatory mechanisms of LSPCs.

## Methods

### Animals

C57BL/6 mice were purchased from Shanghai Laboratory Animal Center to provide fetal livers. No randomization method was used for group allocation, and animals were randomly selected and no animals were excluded from experiments.

### Preparation of mouse liver cells

Liver cells were prepared from E11.5, E12.5, E13.5, E14.5, E16.5 and E18.5 mouse embryos and P2.5 and P3.25 neonatal mice. After adult or neonatal animals were euthanized by CO_2_, fetal livers from siblings of littermates were isolated, pooled, minced and digested in 0.05% collagenase IV (Sigma-Aldrich) at 37 °C for 30 min. After the cells were dissociated by repeated pipetting, the suspensions were filtered through a 40-μm cell strainer to remove undigested tissues. The cells were washed twice with RPMI 1640 medium containing 10% FBS after centrifugation at 300×*g* for 5 min. The cell densities and sizes were determined to select suitable C1 RNA-Seq IFC.

### Single-cell qPCR and RNA-Seq

Single cells with diameters of 10 ~ 17 μm were captured randomly on a C1 RNA-Seq IFC (Fluidigm). A SMARTer Ultra Low RNA kit for Illumina (Clontech) was used for on-chip cell lysis, reverse transcription and cDNA amplification. An ERCC Spike-in Mix (Ambion) was used as the technical control. The quality of cDNA libraries was checked by single-cell qPCR using selected genes on a BioMark HD system (Fluidigm), and the qPCR primers are shown in Additional file 5: Table S4. cDNA were fragmented and prepared using Nextera XT Kit and Index Kit (Illumina). Single-cell libraries were pooled and sequenced by NextSeq 500 (Illumina), with 2 × 151 bp or 2 × 76 bp sequencing modes. To check the expression levels of *Epcam*, single-cell qPCR was carried out using SYBR® Premix Ex Taq™ (Clontech) on the StepOnePlus system (Applied Biosystems).

### Immunofluorescence assay

Fetal livers from E12.5 mouse were embedded in OCT compound, and 5-μm cryostat sections were fixed with 4% paraformaldehyde and then permeabilized with 0.1% Triton X-100. The sections were blocked with 1% BSA in Tris-buffered saline for 1 h at room temperature. To detect co-expression of Dlk1/Hnf4a or Dlk1/vimentin, the sections were incubated with the following primary antibodies overnight at 4 °C: Goat anti-Dlk1 antibody (C-19) (Santa Cruz, PN sc-8624), 1/100; Rabbit anti-HNF-4α antibody (H-171) (Santa Cruz, PN sc-8987), 1/100; Rabbit anti-Vimentin antibody [EPR3776] (Abcam, PN ab92547), 1/250. The sections were washed 3 times with Tris-buffered saline with 0.1% Tween 20 for 5 min each time, and then incubated with the following secondary antibodies for 1 h at room temperature: Donkey anti-Goat IgG (H + L) Cross-Adsorbed Secondary Antibody, Alexa Fluor® 488 (Invitrgen, PN A11055), 1/1000; Donkey anti-Rabbit IgG (H + L) Highly Cross-Adsorbed Secondary Antibody, Alexa Fluor 647 (Life Technologies, PN A31573), 1/1000. The nuclei were counterstained with DAPI, and immunofluorescence was detected with a fluorescent confocal microscope (Nikon).

### Flow cytometric analysis and cell sorting

The following antibodies were used for flow cytometric analysis: E-cadherin, PE-conjugated anti-mouse/human CD324 (E-cadherin) antibody (BioLegend, PN 147304); Anpep, FITC-conjugated anti-CD13 antibody (Abcam, PN ab33486); Dlk1, APC-conjugated mouse Pref-1/DLK1/FA1 antibody (R&D Systems, PN FAB8634A-100); Prom1, PE-conjugated anti-Prominin-1 mouse antibody (Miltenyi, PN 130-102-210); and Epcam, PE-labeled anti-mouse CD326 (Epcam) antibodies (BioLegend, PN 118205). The incubation of fetal liver cells and antibodies were carried out per the vendors’ suggestions. Co-expression analyses of E-cadherin and Anpep, E-cadherin and Dlk1, Anpep and Dlk1, Dlk1 and Prom1 and E-cadherin, and Anpep and Dlk1 were performed using a MoFlo™ XDP cell sorting system (Beckman Coulter). Epcam^+^ cells were sorted from P3.25 fetal liver for single-cell RNA-Seq.

### Sequencing data processing

bcl2fastq2 Conversion Software (Illumina) was used for de-multiplexing, adaptor trimming and generation of FASTQ files for each single cell according to the unique barcode combinations of Nextera XT Index kit. The raw reads in FASTQ format were mapped via Tophat onto the mouse reference genome GRCm38/mm10 downloaded from UCSC Genome Browser, using the default parameters. The transcript levels were then quantified as FPKM values generated by Cufflinks with default parameters. The FPKM values of ERCC Spike-ins were obtained in the same way with ERCC92 reference sequences. The FPKM values of genes and ERCC Spike-ins were then adjusted according the relative proportions of sequencing reads mapped to mouse genome or ERCC92 reference sequences.

### Gene filtering to reduce technical noise

To reduce the interferences in the gene expression-based cell clustering analyses, the transcripts severely corrupted by noise should be removed. Here, we employed a novel approach to identify the noise corrupted transcripts. Specifically, we first constructed a regression model between noise variance and mean transcript RNA-seq FPKMs [34]:

$$ {CV}^2={a}_1/\mu +{a}_0 $$

where *CV* ^2^ *= σ*_*0*_^2^/*μ* ^*2*^, *σ*_*0*_^2^ denotes the noise variance and *μ* denotes the average transcript reads, *a*_*0*_ and *a*_*1*_ are constants. The regression parameters *a*_*1*_ and *a*_*0*_ were estimated by constructing a generalized linear model regression between *CV* ^2^ and 1/*μ*. In the model construction, *CV* ^2^ is approximated by *σ* ^2^/*μ* ^2^, where *σ* ^2^ is the variance of a combination of noise and biological component. The noise variance for a given transcript can be estimated by *σ*_*0*_^2^ = *a*_*0*_ *μ* ^2^+ *a*_*1*_ *μ*. To reduce the uncertainty in model construction, only the transcripts with *μ* > μ_th_ were applied to build the regression model. The *μ*_*th*_ was chosen such that the transcripts with *μ* > *μ*_*th*_ only have 5% with *CV* ^2^ > 0.3. With the estimated noise variance *σ*_*0*_^2^, the reliability of each transcript was evaluated by the following criteria: (1) the 95% confidence interval calculated using *μ* and *σ*_*0*_^2^ should not include zero, which guarantees the overall expression of the given transcript is large enough to surpass noise interference; (2) *σ* ^2^ of the transcript should be larger than the corresponding *σ*_*0*_^2^, which filters out the transcripts which are unlikely to be differentially expressed over the dataset. Only those transcripts fulfilled the two criteria at the same time were retained for further analyses.

### Single-cell data analysis

Outlier identification, HC, principal component analysis (PCA), violin plot and one-way ANOVA of single-cell qPCR and RNA-Seq data were performed using the SINGuLAR™ Analysis Toolset R package (Fluidigm). After gene filtering, the gene expression data of 507 single cells were fed into the identifyOutliers () function of SINGuLAR for outlier filtering with default threshold, and 456 single cells (89.9%) passed the filtering. For the 52 Epcam^+^ cells from P3.25 livers, 10 cells were identified as outliers, and 7 cells were further removed as they showed different gene expression pattern compared with the remaining 35 typical cholangiocytes.

For grouping of all fetal liver cells from E11.5 to E16.5, un-supervised HC of all the liver cells with the top 400 genes ranked by PCA scores was performed and then identified two groups. Among them, one group is hepatoblasts which express both hepatic-related and stem/progenitor-related genes. After removing cells belonging to hepatoblasts, un-supervised HC assigns the remaining cells into six major sample clusters (Sc-1, 2, 3, 4, 5 and 6). Erythrocyte-related genes were not included in the analysis as the cells in the embryonic stages express such genes at relatively low expression level compared with erythrocytes for undetermined reasons. Expression of known markers facilitated the cell type identification of each cluster, where both Sc-1 and Sc-5 cells expressing *Ptprc* (*Cd45*), *Cd68* and *Cd52* are likely to be macrophages, and thus merged as one group. After annotation of putative cell type identification of each single cell, one-way ANOVA was conducted and the top 400 ANOVA-ranked genes were used for the HC plot. Cell clusters were also visualized with t-SNE [35].

For the construction of developmental track of hepatoblasts, the developmental stage information of each single cell was annotated, and only these genes expressed in at least 3 single cells were included in one-way ANOVA to make sure that these genes are indeed related to the liver developmental process.

### Gene ontology (GO) categories and gene set enrichment analysis (GSEA)

In the cell type identification process, the gene sets specifically expressed in each type (Fig. 2a) were analyzed to find out functional terms enriched based on GO Biological Process datasets, and Bonferroni correction for multiple testing was used [36, 37]. The statistically significantly (*p* < 0.05) enriched GO terms are helpful for cell type identification. For pairwise comparison of single cell groups, GSEA [38] was employed to identify the gene sets that are enriched in either group, with the following criteria: *p* < 0.05 and FDR *q* < 0.25. GSEA was carried out for comparison of hepatoblast groups across developmental stage E11.5 ~ E16.5. The detailed definitions of *p* and *q* values and corrections can be found in the references on GO and GSEA [36–38].

### Transcription factor covariance network construction

Transcription factor list of *Mus musculus* from Transcription Factor Database [39] was used to screen TFs that show statistically significantly (*p* < 0.01) differential expression between hepatoblasts and cholangiocytes. Pairwise Pearson correlation coefficients between these selected TFs expressed in single cells were calculated to identify TFs that correlate with at least three other TFs with correlation coefficient higher than 0.35. The matrix was used to construct a weighted network using graph.adjacency () function of igraph package implemented in R as previously described [40], where vertices represent TFs and edges represent high correlation. The networks were visualized with Fruchterman-Reingold layout.

### Performance assessment of the genes for isolation of hepatoblasts

Whether a marker is suitable for isolation of LSPCs can be assessed by two factors: (1) sensitivity, which refers to whether the marker-positive cells include most of the LSPCs; and (2) specificity, which refers to whether the purity of the marker-positive cells excludes other types of cells. The isolation sensitivity and specificity could be defined by randomly selected single cells and cell type information inferred from global transcriptional profiles. For each gene, we calculated its isolation sensitivity and specificity for hepatoblasts, and mesenchymal cells as a comparison. In addition to common isolation markers for hepatoblasts, we also attempted to predict new markers for isolation of hepatoblasts. We first used a cutoff of 0.5 for both sensitivity and selectivity for each group at each stage, found the genes that are shared by different stages, and then selected membrane receptors for further analysis. Plotting the sensitivity vs. specificity across different stages allowed us to analyze whether a given gene marker is suitable for the isolation of hepatoblasts.

## Additional files

Additional file 1: Supplemental figures. **Figure S1.** Single-cell qPCR analysis of mouse fetal liver cells. **Figure S2.** Quality control of single-cell RNA-Seq analysis of mouse fetal liver cells. **Figure S3.** Grouping of fetal liver cells from E11.5 to E16.5. **Figure S4.** Validation of single-cell RNA-Seq results by immunofluorescence and qPCR. **Figure S5.** Dynamic developmental process of mouse LSPCs at single-cell resolution. **Figure S6**. Comparison of the gene expression patterns of some marker genes between mouse and human for cholangiocyte and hepatoblast. **Figure S7.** Assessment and prediction of LSPC biomarkers. **Figure S8.** Validation of some markers for LSPC isolation via flow cytometric analysis. (PDF 2895 kb)

Additional file 2: Table S1. Statistics of RNA sequencing reads of single cells. (XLSX 38 kb)

Additional file 3: Table S2. Top 30 ANOVA ranked genes for E11.5 ~ E16.5 hepatoblast. (XLSX 12 kb)

Additional file 4: Tables S3. Transcription factors differentially expressed between hepatoblast and cholangiocyte for covariance networks. (XLSX 12 kb)

Additional file 5: Table S4. Primers used for single-cell qPCR. (XLSX 11 kb)

## Abbreviations

ANOVA: Analysis of variance
EMT: Epithelial-mesenchymal transition
FPKM: Fragments per kilobase of exon model per million
GO: Gene Ontology
GSEA: Gene set enrichment analysis
HC: Hierarchical clustering
LSPCs: Liver stem/progenitor cells
MET: Mesenchymal-epithelial transition
PCA: Principal component analysis
TFs: Transcription factors
t-SNE: t-distributed stochastic neighbor embedding
