# Supplementary material for: Single-cell RNA-Seq analysis reveals dynamic trajectories during mouse liver development
Source: BMC Genomics. 2017 Dec 4;18:946. doi: 10.1186/s12864-017-4342-x (PMC5715535; doi:10.1186/s12864-017-4342-x)
Supplement: Supplementary file 1 — Supplemental figures. Figure S1. Single-cell qPCR analysis of mouse fetal liver cells. Figure S2. Quality control of single-cell RNA-Seq analysis of mouse fetal liver cells. Figure S3. Grouping of fetal liver cells from E11.5 to E16.5. Figure S4. Validation of single-cell RNA-Seq results by immunofluorescence and qPCR. Figure S5. Dynamic developmental process of mouse LSPCs at single-cell resolution. Figure S6. Comparison of the gene expression patterns of some marker genes between mouse and human for cholangiocyte and hepatoblast. Figure S7. Assessment and prediction of LSPC biomarkers. Figure S8. Validation of some markers for LSPC isolation via flow cytometric analysis. (PDF 2895 kb) [file 12864_2017_4342_MOESM1_ESM.pdf]

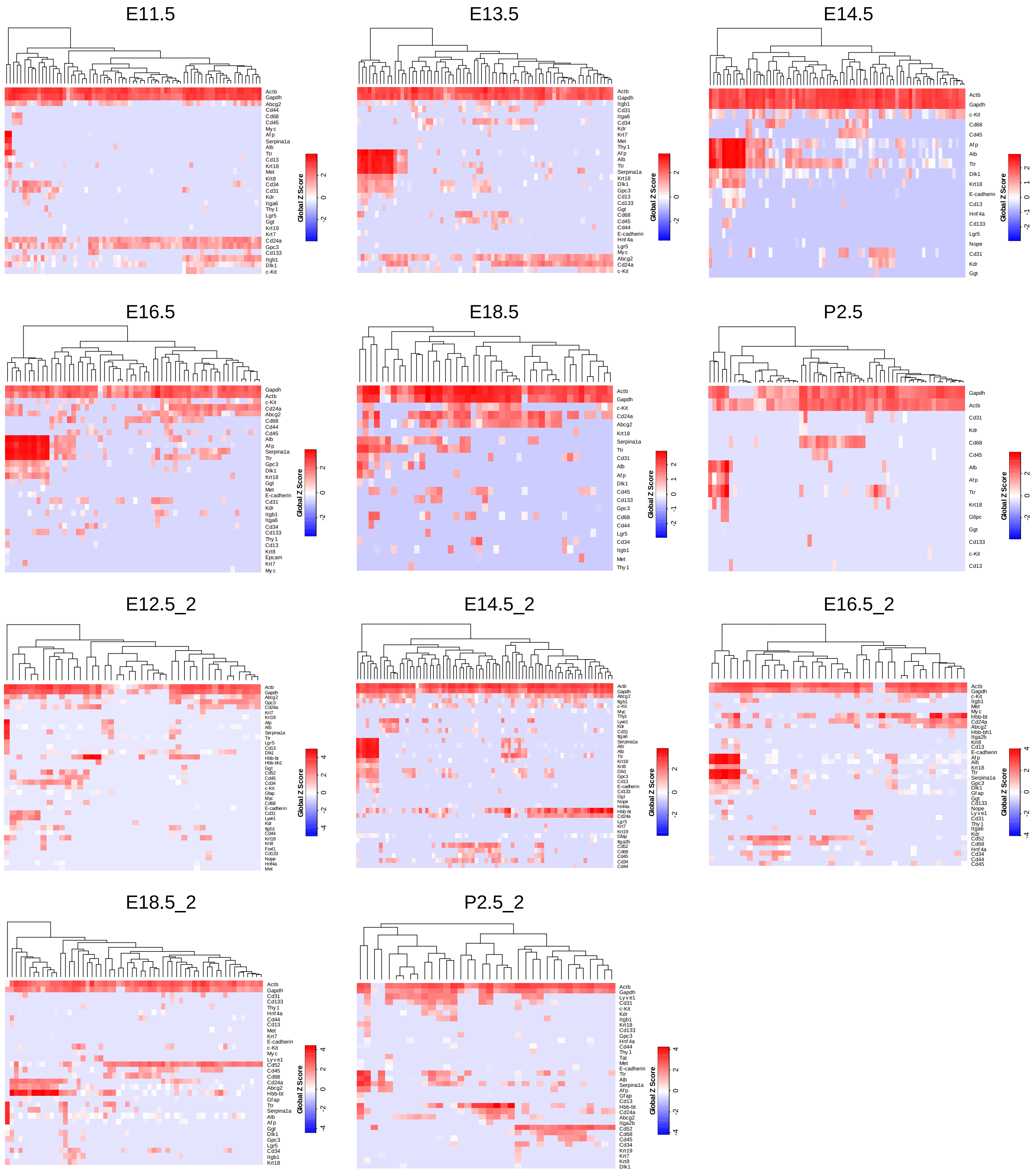

Figure S1 Single-cell qPCR analysis of mouse fetal liver cells. The first batch included the following developmental stages: E11.5, E12.5 (shown in Fig. 1c), E13.5, E14.5, E16.5, E18.5 and P2.5; another batch (labeled as _2) of E12.5, E14.5, E16.5, E18.5 and P2.5 was also analyzed.

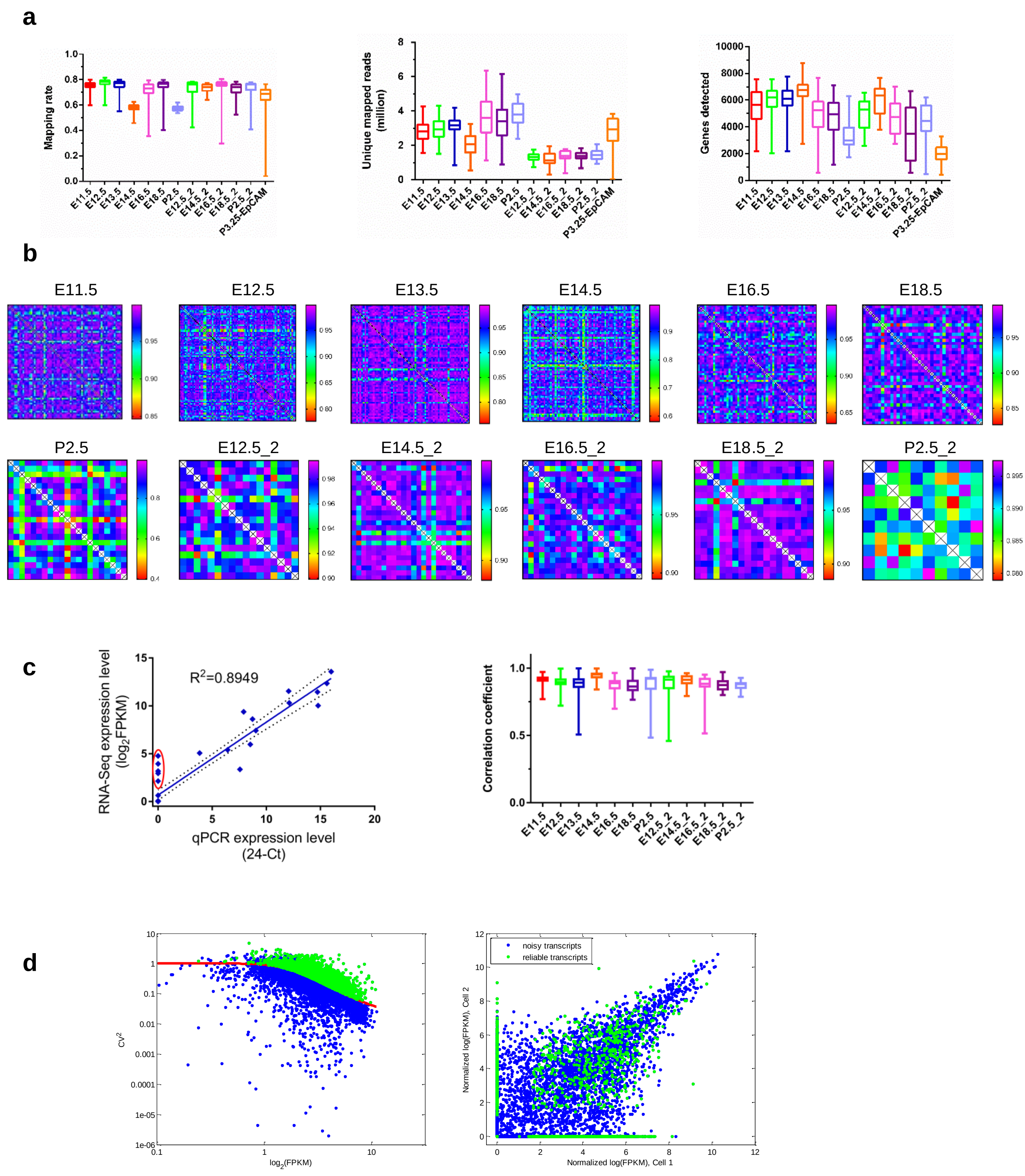

Figure S2 Quality control of single-cell RNA-Seq analysis of mouse fetal liver cells. **a** Box-and-whisker plots showing the statistics of the single-cell RNA-Seq data. **b** Heat-map showing the Pearson correlation coefficients between single cells within the same developmental stage based on the 92 ERCC Spike-ins. **c** Correlation between single-cell RNA-Seq and qPCR. One representative cell is shown on the left panel, with dotted lines indicating the 95% confidence interval for the regression line. The genes highlighted in red circle were detected by single-cell RNA-Seq but not qPCR. Distributions of the Pearson correlation coefficients between single-cell RNA-Seq and qPCR for different stages are shown on the right panel. **d** Gene filtering to reduce technical noise. Random noise fit and reliable transcript identification are shown with the CV^2 against the mean log read counts. The gene expression correlation of two representative single cells is shown, with green dots indicating the reliable genes and blue dots representing the noise-corrupted genes.

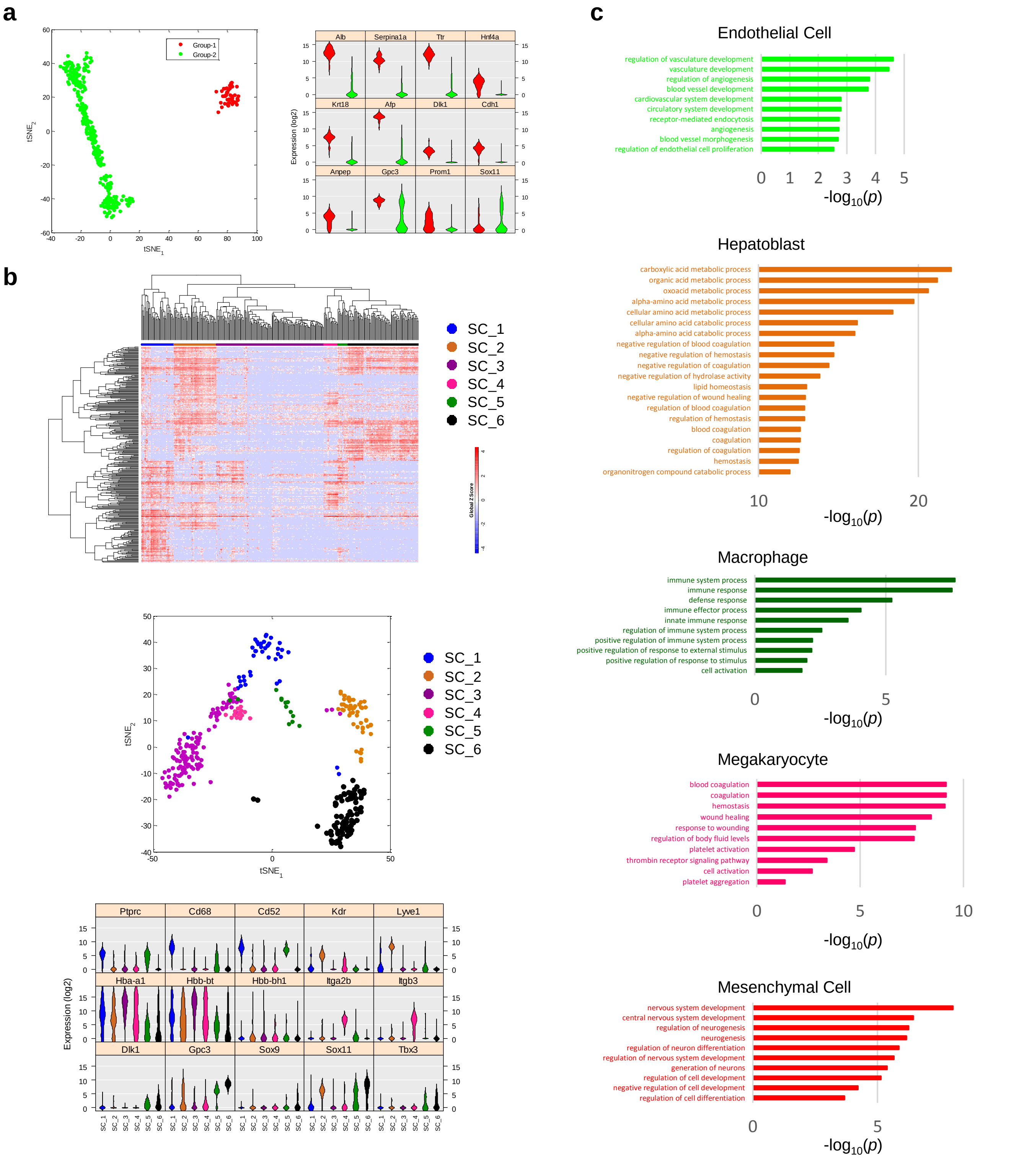

Figure S3 Grouping of fetal liver cells from E11.5 to E16.5. **a** PCA of all the cells with the top 400 genes identified two major groups as shown by t-SNE plot (left), and expression of known markers showed that they were hepatoblasts and non-hepatoblasts (right). **b** Un-supervised HC of non-hepatoblasts with the top 400 genes (excluding erythrocyte-related genes) identified six major sample clusters (Sc) (Sc-1, 2, 3, 4, 5, and 6), and the clusters were shown by t-SNE plot. Violin plot of some marker genes facilitated identification of the cell types. **c** GO enrichment analysis of gene sets specifically expressed in each cell type. The top 10 items enriched in endothelial cells, macrophages, megakaryocytes and mesenchymal cells, and the top 20 items in hepatoblasts were shown.

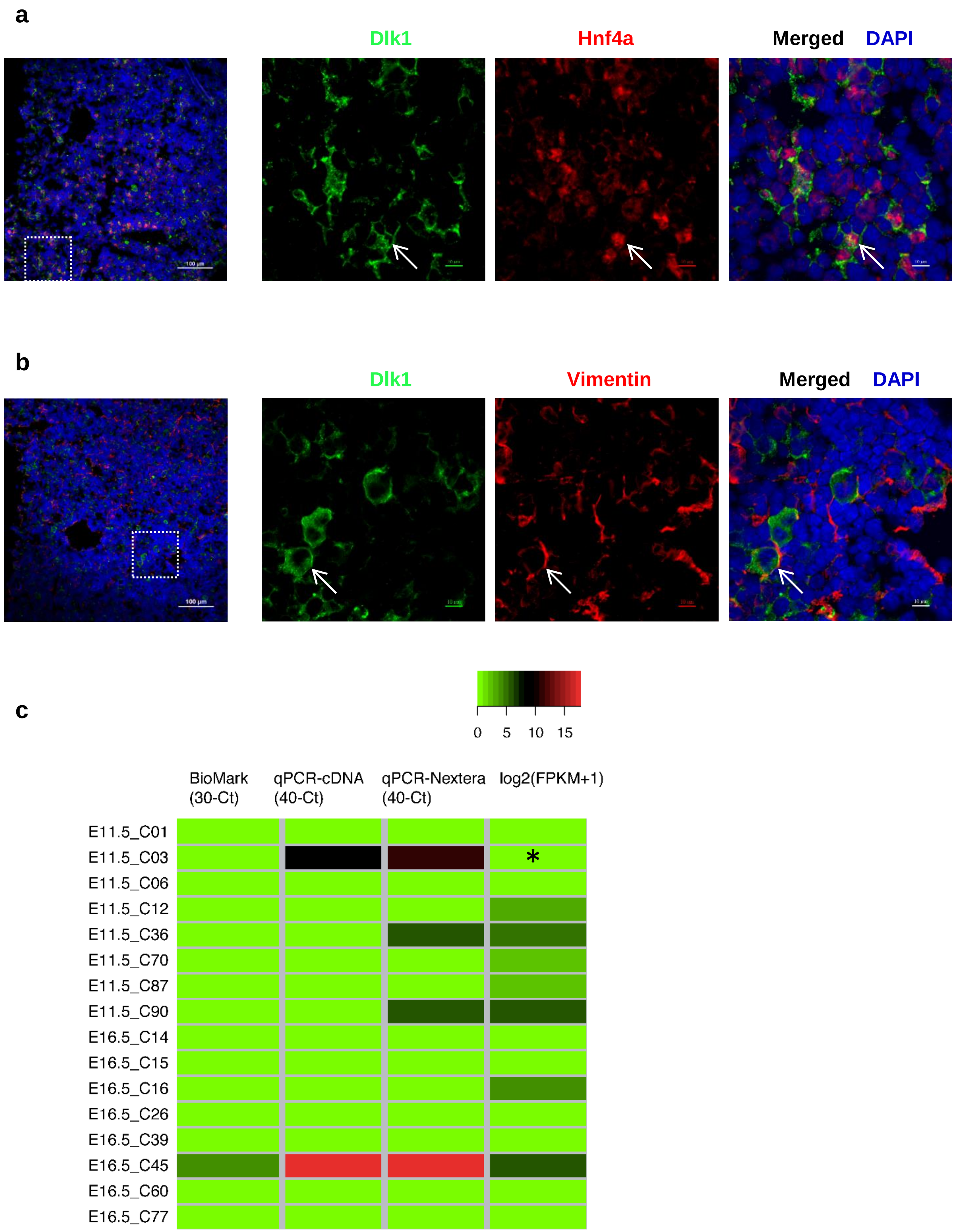

Figure S4 Validation of single-cell RNA-Seq results by immunofluorescence and qPCR. **a** A liver section from E12.5 mouse was co-stained with antibodies against Dlk1 and Hnf4a, and the nuclei were stained with DAPI. The white dotted square in the left panel was shown at higher magnification on the right. A cell showing co-expression of Dlk1 and Hnf4a was shown with white arrow. Scale bar, 100 μm for the left panel and 10 μm for the right panels. **b** Expression profiles of Dlk1 and vimentin in E12.5 mouse liver, similarly presented as **a**. **c** Validation of *Epcam* expression in some single cells by qPCR. For BioMark qPCR, there was no replicate; for tube-based qPCR, data shown were the means of triplicates. '*' indicated there was FPKM value which was too low to be discriminated from the background.

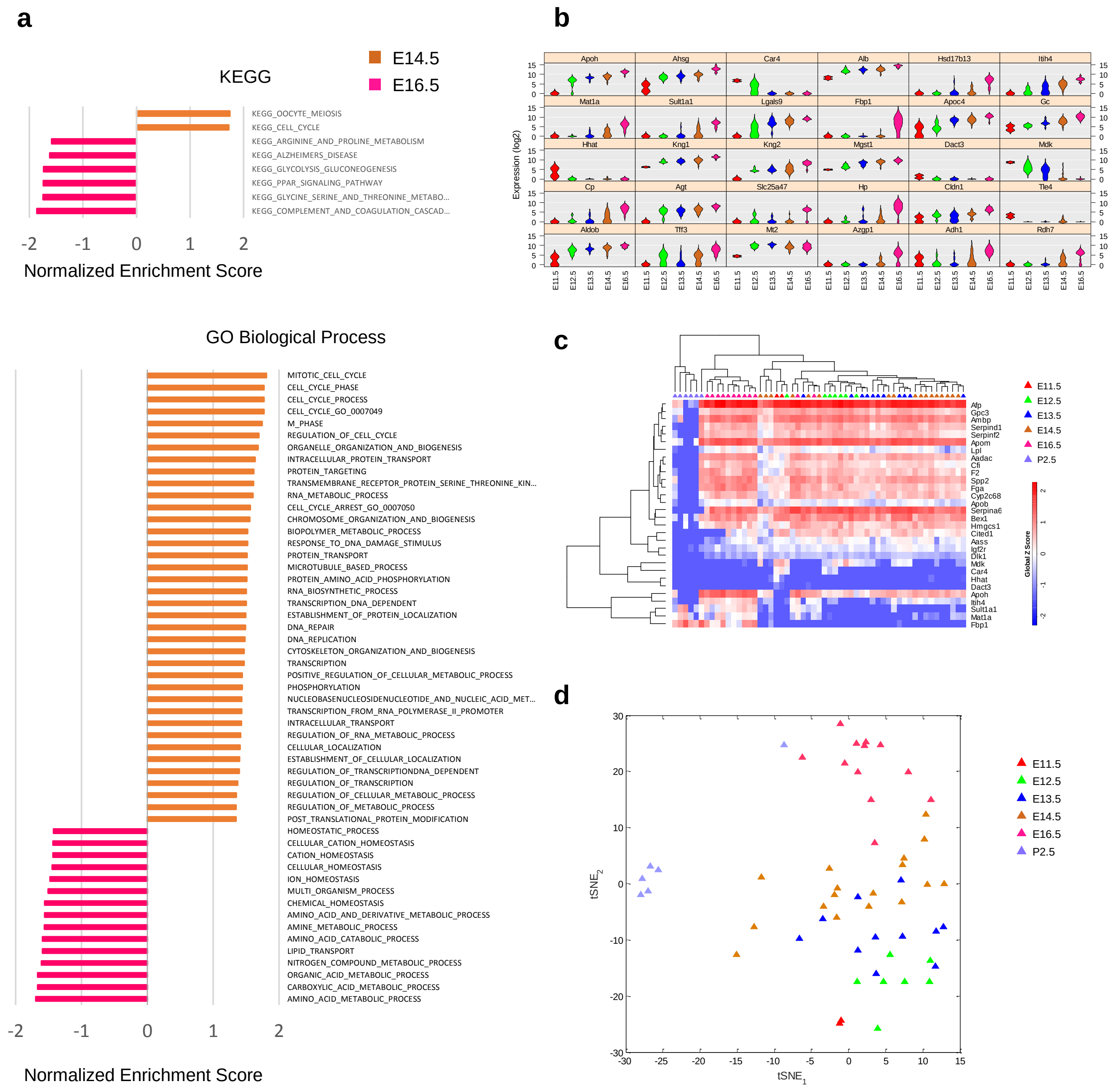

Figure S5 Dynamic developmental process of mouse LSPCs at single-cell resolution. **a** Identification of gene sets enriched in E14.5 or E16.5 hepatoblasts using GSEA. The gene sets enriched in E14.5 or E16.5 hepatoblasts are shown according to datasets of KEGG or GO Biological Process with $p < 0.05$ and FDR $q < 0.25$. **b** Violin plot of the genes related to dynamic developmental process of mouse LSPCs. The top 30 genes that are differentially expressed among the five developmental stages are shown. **c-d** Comparison of embryonic hepatoblasts with hepatic cells from P2.5 mouse livers via HC and t-SNE.

a

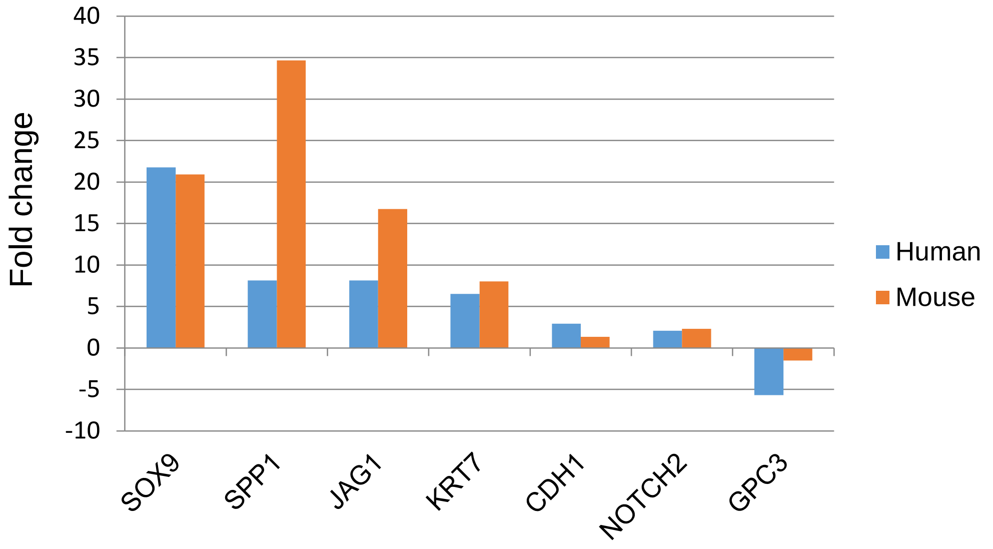

b

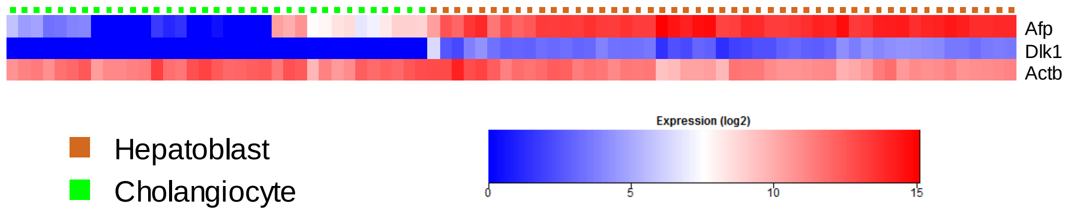

Figure S6 Comparison of the gene expression patterns of some marker genes between mouse and human for cholangiocyte and hepatoblast. **a** Fold change patterns between hESC-Chol and hESC-HB (Dianat et al., Hepatology, 2014, 60(2): 700-714), and between the average values of mouse cholangiocyte and hepatoblast single cells for seven genes. **b** The gene expression pattern of *Afp* and *Dlk1* in mouse single cells of cholangiocyte and hepatoblast, with *Actb* as positive control. As *Dlk1* is not expressed in cholangiocytes, it is included in **b** rather than **a**.

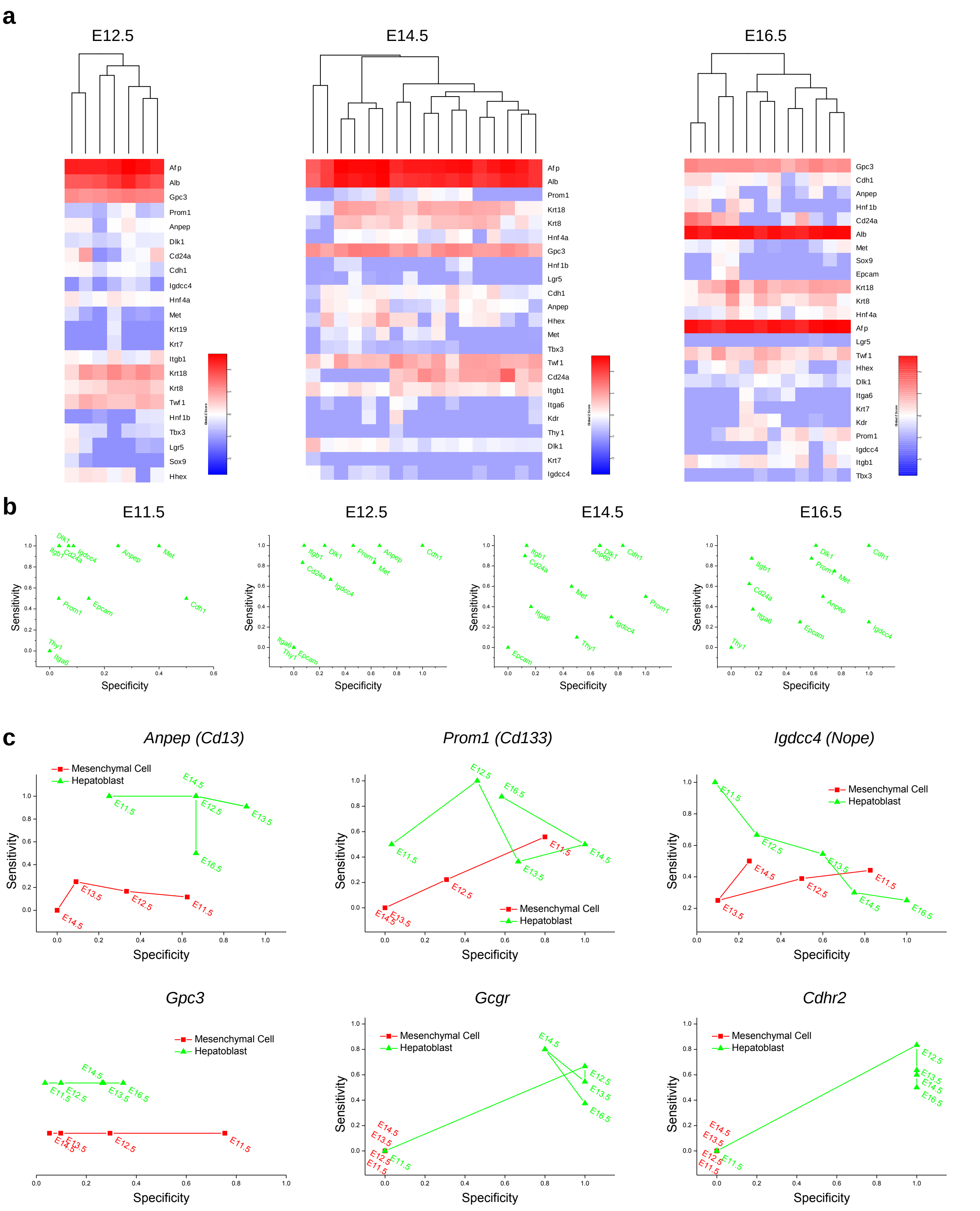

Figure S7 Assessment and prediction of LSPC biomarkers. **a** Heterogeneity of the transcriptional expression of some selected marker genes in LSPCs, as shown by HC analysis of the selected marker genes in hepatoblasts from different developmental stages. **b** Sensitivity *vs.* specificity plot of cell isolation using 11 selected markers for hepatoblasts at E11.5, E12.5, E14.5 and E16.5. **c** Sensitivity and specificity of LSPC isolation using four known marker genes, *Anpep*, *Prom1*, *Igdcc4* and *Gpc3*, and two predicted gene markers, *Gcgr* and *Cdhr2*, across the five developmental stages. For *Gpc3*, the sensitivity for the two groups was equal to 1, so the two lines were manually relocated in the *y*-axis for better visualization.

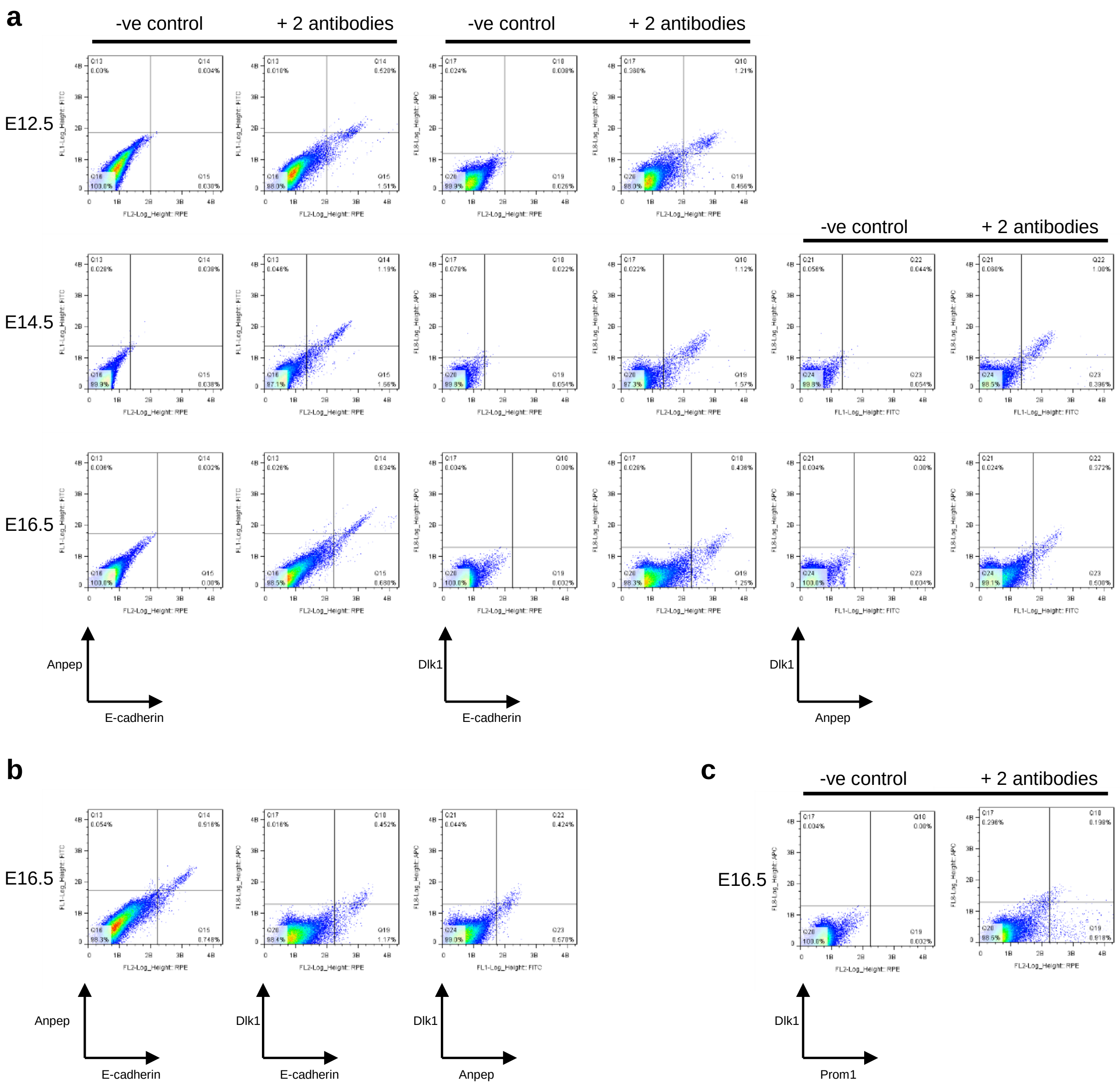

Figure S8 Validation of some markers for LSPC isolation via flow cytometric analysis. **a** Co-expression analysis of E-cadherin and Anpep, E-cadherin and Dlk1, and Anpep and Dlk1 in mouse fetal livers of different developmental stages. **b** Co-expression analysis of E-cadherin, Anpep and Dlk1 in E16.5 mouse fetal livers. **c** Co-expression analysis of Dlk1 and Prom1 in E16.5 mouse fetal livers. Representative images from two replicative reactions for each condition are shown, and reactions without antibodies were used as negative controls.
